# Research on the influence of polar solvents on CsPbBr_3_ perovskite QDs†

**DOI:** 10.1039/d1ra04485k

**Published:** 2021-08-10

**Authors:** Yifang Sun, Huidan Zhang, Kai Zhu, Weiguang Ye, Lushuang She, Ximing Gao, Wenyu Ji, Qinghui Zeng

**Affiliations:** State Key Laboratory of Luminescence and Applications, Changchun Institute of Optics, Fine Mechanics and Physics, Chinese Academy of Sciences Dong_Nanhu Road 3888 Changchun 130033 P. R. China qhzeng@ciomp.ac.cn; University of Chinese Academy of Sciences Beijing 100049 P. R. China; Innovation Practice Center, Changchun University of Chinese Medicine Changchun 130017 P. R. China; Key Lab of Physics and Technology for Advanced Batteries (Ministry of Education), Department of Physics, Jilin University Changchun 130012 P. R. China

## Abstract

All-inorganic CsPbX_3_ (X = Cl, Br, I) perovskite quantum dots (QDs) have become a kind of optoelectronic material with huge application prospects due to their excellent physical and optical properties. However, their poor structural stability to the external environment, especially polar solvents, seriously hinder further development in practical applications. Considering whether polar solvents have the same effects on perovskites QDs, few studies have been investigated in this area presently. In order to find out the effect of different polar solvents on all-inorganic perovskite QDs, in this work, we select 12 kinds of polar solvents of methanol, ethanol, isopropanol, 1-butanol, 1-pentanol, 1-octanol, *N*,*N*-dimethylformamide (DMF), tetramethylethylenediamine (TMEDA), ethyl acetate, *n*-butyl acetate, dibutyl phthalate and acetone for a specific analysis. The characterization of their morphology, optical and physicochemical properties shows that different polar solvents have different effects on all-inorganic perovskite QDs, but their effects are regular. Polar solvents act on the ligands preferentially, and the effects can be divided into: reducing the concentration of ligands; substituting ligands partially; completely destroying the surface ligands; polar solvents with the same functional group, as the polarity of the solvent increases, the impact on all-inorganic perovskite QDs is greater. We believe that this discovery has important implications for improving the stability of all-inorganic perovskite QDs.

## Abstract 2

All-inorganic CsPbX_3_ (X = Cl, Br, I) perovskite quantum dots (QDs) have become a kind of optoelectronic material with huge application prospects due to their excellent physical and optical properties.

## Graphical abstract

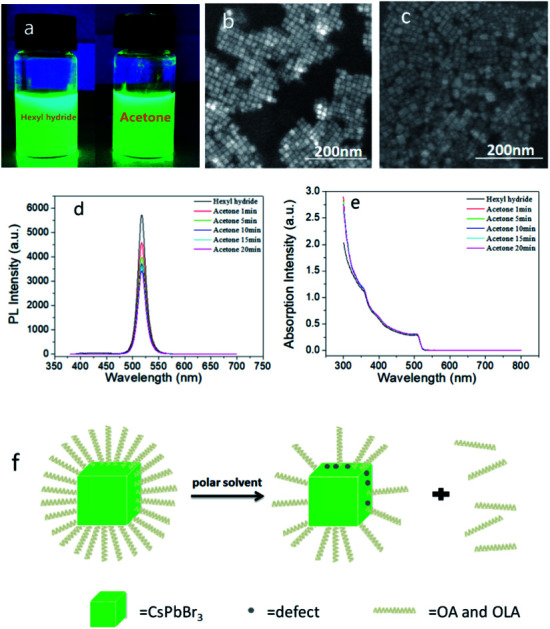

## 1. Introduction

All-inorganic CsPbX_3_ (X = Cl, Br, I) perovskite quantum dots (QDs) have become a kind of photoelectric material which possess great potential application due to their unique optical versatility, such as efficient luminescence, narrow emission spectrum properties, high defect tolerance, adjustable emission wavelength, high absorption coefficient, and long diffusion length of carrier.^1–4^ As future optoelectronic materials, all-inorganic CsPbX_3_ perovskite QDs (PQDs) have attracted the intensive attention of diverse materials scientists in recent years. In 2015, Kovalenko *et al.* reported a new avenue to synthesize CsPbX_3_ PQDs which needed high temperature and inert gas protection.^5^ In 2016, Zeng *et al.* developed a new method to synthesize CsPbX_3_ at room temperature.^6^ With the development of CsPbX_3_ PQDs, they have been widely applied in optoelectronics research field, such as light-emitting diodes (LEDs),^7^ photodetectors,^8–10^ solar cells,^11,12^ and single-photon emitters.^13^ However, further development towards practical applications is severely hindered by their poor structural stability against external environment.^14–16^

Due to their inherent ionic nature, all-inorganic CsPbX_3_ PQDs are extremely sensitive to polar solvents, which usually make them lose their optical properties, surface ligands and even structural integrity.^17^ In general, the source of their instability is usually attributed to their low formation energy, which makes the properties of these materials vulnerable to external environmental influences.^18,19^ Moreover, environment can affect the existence state of ligand molecules, and the ligand molecules can affect the properties of CsPbX_3_ PQDs. It is commonly believed that, the ligands are in harmony with its surface, in addition to the electronic structure, it also affect the chemical and colloidal stability of QDs.^20^ Compared with classic chalcogen QDs, the ionic characteristics of CsPbX_3_PQDs are more obvious, and the interaction with the surface ligands are also more ionic and unstable.^21^ Current research suggests that the characteristics of ionic bonding and lower lattice energy result in a fact that CsPbX_3_ PQDs are almost soluble in all polar solvents.^20^ However, the polar solvents cause the ligands to be desorbed progressively, which lead to the exposure of CsPbX_3_ PQDs to polar solvents ultimately, and CsPbX_3_ PQDs will be quickly decomposed, and lose its colloidal stability and structural integrity eventually.^22^

Polar solvents have a great influence on CsPbX_3_ PQDs: such as luminescent properties, morphological characteristics, *etc.* However, up to now, few studies have been reported on the specific principle of action between different polar solvents and PQDs, the different effects of polar solvents, or whether the final effects of polar solvents are the same. We believed that this is really important for this work, and a clear study of the law of action of polar solvents has instructive significance on how to improve the solvent stability of PQDs. Here, twelve polar solvents of methanol, ethanol, isopropanol, 1-butanol, 1-pentanol, 1-octanol, *N*,*N*-dimethylformamide (DMF), acetone, ethyl acetate, *n*-butyl acetate, dibutyl phthalate and tetramethylethylenediamine (TMEDA), were selected to analyze the action mechanism. In this work, all-inorganic CsPbX_3_ PQDs were directly synthesized at high temperature *via* the hot injection technique,^23^ and CsPbX_3_ PQDs were characterized by field emission scanning electron microscopy (FE-SEM, Hitachi, S-4800), energy dispersive spectrometer (EDS), UV-3101 spectrophotometer, Hitachi F-7000 fluorescence spectrofluorometer and ultraviolet (UV) light, to find out the action law of different polar solvents. Finally, comparing the experimental data, we believed that the influence of polar solvents on CsPbX_3_ PQDs can be divided into four categories: 1. competing for ligands, resulting in new defects due to lack of ligands; 2. partial replacement of oleic acid (OA) and oleylamine (OAM) ligand molecules by TMEDA, changing the morphologic characteristics and luminescence stably; 3. destroying the light-emitting structure completely and further aggregated into large particles finally; 4. the influence of alcohols on all-inorganic PQDs is related to the order of polarity: the stronger the polarity were, the faster the PQDs quenched, and the more the photoluminescence quantum yield (PLQY) decreased.

## 2. Results and discussion

### 2.1 Competing for ligands, resulting in new defects due to lack of ligands

**Fig. 1:**
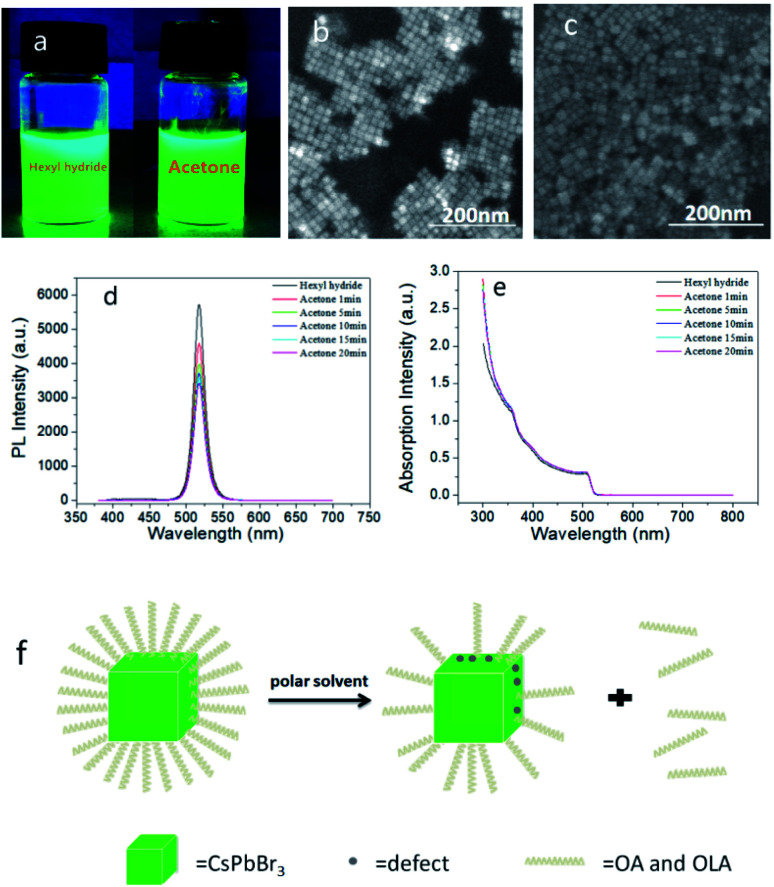
Photograph of the sample A and sample B1 under UV light (a). FE-SEM images of sample A (b) and sample B1 (c). The PL (d) and absorption (e) spectra of sample B1 over time. Schematic diagram of the mechanism of action when acetone was added to the PQDs solutions (f).

Firstly, we added 50 μl PQDs with a concentration of 21 mg ml^−1^ in 6 ml *n*-hexane solution and named it as sample A, the solution added 50 μl acetone additionally, named as sample B1, added 50 μl ethyl acetate additionally named as sample B2, added 50 μl *n*-butyl acetate additionally named as sample B3, and added 50 μl dibutyl phthalate additionally named as sample B4. As shown in Fig. 1a and ES1,† B1–B4 still had a high brightness under UV light, and the brightness was slightly reduced compared with A. By observing the SEM images (shown in Fig. 1b, c and ESI S2†), we found that the small particles of sample B1–B4 were distributed uniformly, with clear particle morphology and presented cubic shape. We believed that the cubic structure of the quantum dots has not been destroyed. In terms of the PL (Fig. 1d and ESI S3†) and absorption property (Fig. 1e and ESI S3†), the peaks of the PL spectra gradually decreased with time, and stabilized after 10 minutes. Additionally, sample B1–B4 had a clear excitonic absorption peak, the PL peak wavelength of sample A was 517.4 nm, the peak of sample B1 was 517.8 nm (Fig. 1d), while the peaks of B2–B4 were almost unchanged (ESI S3†). As we all know, the full width at half maximum (FWHM) could reflect the size and shape distribution of PQDs, the narrower the FWHM was, the more uniform the PQDs were. Herein in Fig. 1d, the FWHM of sample A and B1 is 17.4 nm and 17.5 nm, while the FWHM of B2–B4 changed less than 0.5 nm respectively, which also proved efficiently that there was almost no change in particle size and the homogeneity of the PQDs. The PLQY decreased from 90% initially to 68% after 1 minute, 59% after 5 minutes, 55% after 15 minutes, 52% after 20 minutes, and 51% after 25 minutes, while sample B2 reduced to 87.58%, sample B3 reduced to 70.34%, sample B4 reduced to 71.48% (ESI S4†). In summary, after adding B1–B4, the cubic structure of PQDs had not been destroyed, and still had a high brightness, however, the PLQY was reduced. Therefore, we believed that ethyl acetate, *n*-butyl acetate, dibutyl phthalate and acetone competed with PQDs for ligands, which contributed to OA and OAM ligand molecules' falling off partially, besides, the detachment of binding ligands would lead to the exposure of surface dangling bonds, introducing an increase in the number of defect trap states.^24^ As a result, new defects were generated, non-radiative recombination increased, and affected the luminescence characteristics, and the PLQY was greatly reduced ultimately. Due to the reduction of ligands and the existence of defects, the long-term reaction would also produce aggregation, but the fluorescence effect were still maintained. The specific mechanism of action is clearly shown in Fig. 1f.

### 2.2 Partial replacement of OA and OAM ligands by TMEDA, changing the morphologic characteristics and luminescence stably

**Fig. 2:**
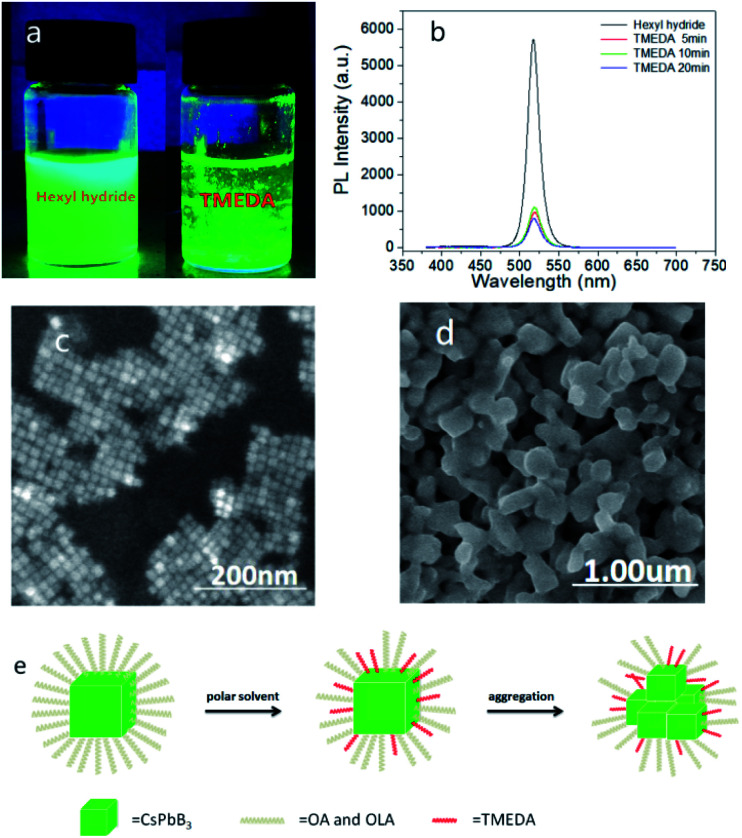
Photograph of sample A and sample B5 under UV light (a). The PL spectra of sample B5 over time (b). FE-SEM images of sample A (c) and sample B5 (d). Schematic diagram of the reaction mechanism with PQDs after adding TMEDA (e).

In this work, we added 50 μl TMEDA in sample A and named it as sample B5. After dissolving TMEDA in the solution, the white floccule precipitates appeared rapidly, showing high brightness under UV lamp (Fig. 2a) and almost constant brightness after standing for several months. Sample B5 still had fluorescence reaction under UV lamp, which meant that the effective light-emitting structure had not been destroyed completely. Surface ligands would affect the dispersion state of PQDs, after adding TMEDA, the aggregation of PQDs indicated that the surface ligands had changed. Subsequently, the flocculent white precipitate was dropped on the silicon wafer, and dried in the shade at room temperature. The PL spectra were shown in Fig. 2b. Comparing with sample A, the PL peak of sample A was located at 517.4 nm, and that of sample B5 was located at 520 nm, while the FWHM increased from 17.4 nm to 18.6 nm. Then, combined with the SEM images, sample A shown typical cubic structure while sample B5 shown inhomogeneous morphologies, which proved that TMEDA promoted the aggregation and precipitation of PQDs, and the particle size increased (Fig. 2c and d). Moreover, comparing the EDS diagram of sample A, after reacting with TMEDA, the content of N element increased, while the content of O element decreased (ESI, diagram S5†), where N element could stand for TMEDA and O for OA and OAM. After reacting with TMEDA, the content of OA and OAM ligands in PQDs decreased while the content of TMEDA increased, thus, we concluded that TMEDA replaced OA and OAM long chain ligands partially. As was well-known, surface ligands played a vital role in separating colloidal PQDs, which could prevent them from irreversibly aggregating caused by high surface activity of nano-sized particles.^24^ After replacing the OA and OAM ligands by TMEDA partially, the original state of the ligands changed, breaking the equilibrium, which changed the morphology and solubility properties of PQDs, thereby promoting the aggregation and precipitation. However, the effective light emission of PQDs had not been destroyed completely, and there was still strong fluorescence after a few months. The specific mechanism of action is clearly shown in Fig. 2e.

### 2.3 Destroying the light-emitting structure completely and further aggregated into large particles finally

**Fig. 3:**
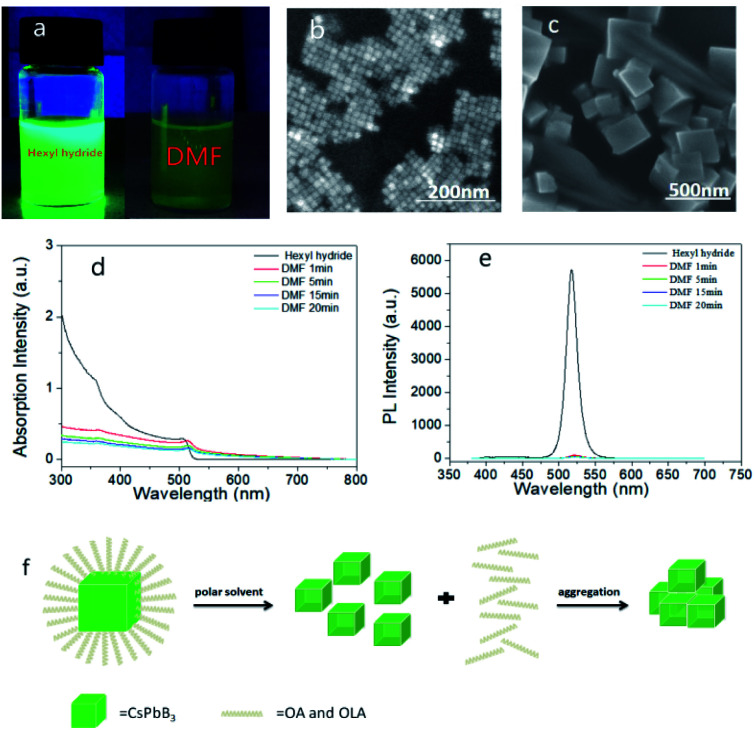
Photograph of sample A and sample B6 under UV light (a). FE-SEM images of sample A (b) and sample B6 (c). The absorption (d) and PL (e) spectra of sample B6 over time. Schematic diagram of the mechanism of action when DMF was added to the PQDs solutions (f).

We added 50 μl DMF in sample A and set it as sample B6. DMF reacted with PQDs quickly, when DMF was added, the brightness dips rapidly accompanied by a color change from green to yellow, and the yellow precipitates only had weak fluorescence under UV light (Fig. 3a). Contrasting to sample A, the SEM image of sample B6 showed large particles (Fig. 3b and c), according to the surface effect, the dangling bonds would be formed when the ligands fell off, and the nanoparticles were very easy to contact with each other, filling the dangling bonds and agglomerated, and then being coalesced into larger particles. In addition, both the absorption and PL spectra dropped instantly (Fig. 3d and e), and the PL peak had a red-shift from 517.4 nm to 521.8 nm, while the PLQY reduced to 3.2% (Fig. S4†). The red-shift in the PL spectra might be due to the increased PQDs size, and the ligands fell off, new defects were generated, non-radiation recombination increased, which led to a decrease in PLQY ultimately. Thus, we believed that in the reaction process, DMF acted on the ligands firstly, caused the surface ligands to fall off, and the effective light-emitting structure of PQDs that losing the protection of the ligands would be destroyed and condensed into large-particle molecules gradually. The specific mechanism of action is shown in Fig. 3f.

### 2.4 The effect of alcohols on PQDs are related to polarity

**Fig. 4:**
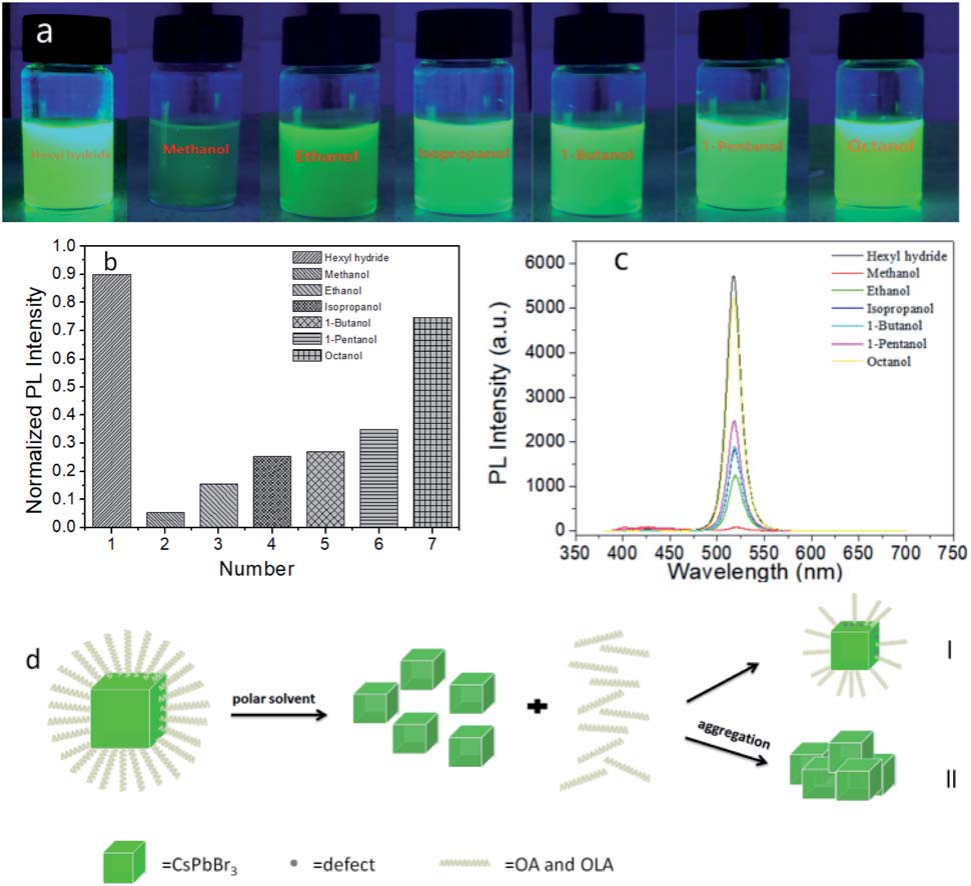
Photograph of sample A and sample B7–12 under UV light (a). The PLQY of sample A and sample B7–12 (b).The PL spectra of sample B7–12 over time (c). Schematic diagram of the reaction mechanism with PQDs after adding alcohols (d), 4d-I represents weakly polar alcohols, and 4d-II represents alcohols with strong polarity.

We added 50 μl methanol, ethanol, isopropanol, 1-butanol, 1-pentanol, 1-octanol to the sample A solution respectively, and set them as sample B7–12 in turn. The polarity of a solvent was related to its dielectric constant, the greater the dielectric constant was, the stronger the polarity was, according to the order of permittivity (ESI, Table 1†), the order of polarity is methanol, ethanol, isopropanol, 1-butanol, 1-pentanol, 1-octanol in sequence. Compared the luminosity of the photos under the UV lamp, we observed that as the polarity of alcohols decreased, the degree of PQDs quenching decreased successively from methanol to 1-octanol, as a result, the fluorescence brightness increased gradually (Fig. 4a). At the same time, the PLQY increased when alcohol polarity decreased (Fig. 4b), PLQY largely depended on the characteristics of the NC surface,^21^ and the surface characteristics were affected by surface ligands, once the ligand balance was broken, it would further affected the PLQY. As the polarity of alcohols increased, the degree of PLQY reduction had been greatly reduced, proving that the polar alcohols had a greater impact on the ligands. Additionally, the reaction of PQDs with alcohols could produce Stokes shift, observing the absorption and PL spectra, we could find that the weaker the polarity was, the smaller the red shift of the emission peak was (Fig. 4c, ESI Table 2†). Comparing the FE-SEM images, the boundaries between particles of sample B*x* (*x* = 7–12) (ESI S7†) are blurred, and small particles stuck together, that was because surface ligands could not only improve the stability of PQDs effectively, but also improve the dispersion of PQDs, the more lack of ligands, such as methanol, PQDs were easier to agglomerate, lower stability, and the morphology also changed. Therefore, we believed that it was caused by OH– group which destroyed the integrity of OA and OAM ligands. Alcohols with weak polarity have poor ability to destroy ligands, which reduced the concentration of ligands, resulting in lower PLQY, but still maintained the structural integrity of PQDs (the specific mechanism of action is shown in Fig. 4d-I). Withal, as the polarity of the alcohol increased, the integrity of the ligands would be destroyed more quickly, and even formed non-fluorescent particles directly if the polarity of alcohols was strong enough. The specific mechanism of action is shown in Fig. 4d-II. In summary, we could draw the conclusion that as the polarity of alcohols increased, their influence on PQDs also increased gradually.

## 3 Conclusions

Different polar solvents have different principles of action on PQDs, the influence needs to consider both polarity and main functional groups (the influences of the used polar solvents have shown in ESI Table 3†). The effect of different functional groups on PQDs has nothing to do with the order of polarity, the polarity of acetone is stronger than that of isopropanol, but the effect on PQDs is weaker. When polar solvents owning with same functional groups reacts with PQDs, as the polarity increases, the destruction ability also increases. Polar solvents act on the surface ligands firstly and destroy the equilibrium, resulting in the removal of ligand molecules, forming new defects and reducing the PLQY eventually. DMF and methanol will cause a large number of ligands to fall off, and due to factors such as surface effects, they will coalesce to form large particles, the light-emitting structure is completely destroyed, and no fluorescence reaction will occur. Acetone, ethyl acetate, *n*-butyl acetate, dibutyl phthalate causes a result that PQDs will lose part of its surface ligands, and becomes stable after PLQY reduced to a stable value. TMEDA partially replaces the OA and OAM long-chain ligands, destroying the equilibrium state of the ligands, and the changes in the ligands will lead to different morphologies, in addition TMEDA can still protect the effective light-emitting structure after replacing the ligands, it can be stored for several months and still have fluorescence. The influence of alcohols is related to the order of polarity, the stronger the polarity is, the greater the destructive power is, the faster the quenching of PQDs are. Among them, TMEDA has the most special influence. We believe that this analysis can provide new ideas for improving the stability of PQDs in polar solvents.

## Conflicts of interest

There are no conflicts to declare.

## Supplementary Material

RA-011-D1RA04485K-s001
