# Supplementary material for: Research on the influence of polar solvents on CsPbBr3 perovskite QDs
Source: RSC Adv. 2021 Aug 10;11(44):27333–7. doi: 10.1039/d1ra04485k (PMC9037818; doi:10.1039/d1ra04485k)
Supplement: RA-011-D1RA04485K-s001 [file RA-011-D1RA04485K-s001.pdf]

Electronic Supplementary Material (ESI) for RSC Advances.
This journal is © The Royal Society of Chemistry 2021

# Electronic Supplementary Information

# Research on the influence of polar solvents on $CsPbBr_3$ Perovskite QDs

Yifang Sun,[a, b] Huidan Zhang,[a, b] Kai Zhu,[c] Weiguang, Ye,[a] Lushuang She,[a, b] Ximing Gao,[a, b] Wenyu Ji,[c] Qinghui Zeng,*[a]

[a] State Key Laboratory of Luminescence and Applications, Changchun Institute of Optics, Fine Mechanics and Physics, Chinese Academy of Sciences, Dong_Nanhu Road 3888, Changchun 130033, P. R. China

E-mail: qhzeng@ciomp.ac.cn

[b] University of Chinese Academy of Sciences, Beijing 100049, P. R.China

[c] Innovation Practice Center, Changchun University of Chinese Medicine, Changchun 130017, P. R. China.

[d] Key Lab of Physics and Technology for Advanced Batteries (Ministry of Education), Department of Physics, Jilin University, Changchun 130033, P. R. China

## Starting materials

Cesium carbonate (99.9%), oleic acid (≥99%), 1-octadecene (90%), oleylamine (≥98%), lead bromide (99.999%) were purchased from Sigma-Aldrich. ethyl acetate (≥99.9%), n-Butyl acetate (≥99%), dibutyl phthalate (≥99%) were purchased from Aladdin. Toluene (≥95%), hexane (≥95%), methanol (≥99.5%), ethanol (≥99.7%), isopropanol (≥99.7%), 1-butanol (≥99%), 1-pentanol (≥95%), 1-octanol (≥99%), N,N-dimethylformamide (≥99.5%), acetone (≥99.5%), and tetramethylethylenediamine (≥99%) were purchased from Beijing Chemical Works. All the reagents were used without further purification.

## Experiment

Dissolved 50μl PQDs with a concentration of 21mg/ml in 6ml n-hexane solution and set it as sample A, the solution added 50 μl acetone additionally was set as sample B1, added ethyl acetate, n-Butyl acetate, dibutyl phthalate separately were set as B2-B4， added 50 μl TMEDA in sample A, were set as sample B5, successively, added 50 μl DMF, set as sample B6, added methanol, ethanol, isopropanol, 1-butanol, 1-pentanol, 1-octanol to the sample A solution, and set them as sample B7-B12 in turn. Shaked sample A and Bx(x=1-12) together evenly with a ball machine, and let them stand for 30 minutes finally.

| Solvent | DMF | methanol | ethanol | acetone | isopropanol | 1-butanol |
|---|---|---|---|---|---|---|
| Permittivity ($C^2/(N \cdot M^2)$) | 36.7 | 32.5 | 24.3 | 20.7 | 18.3 | 17.1 |
| | 1-pentanol | 1-octanol | Ethyl acetate | n-Butyl acetate | dibutyl phthalate | |
| | 13.9 | 10.34 | 6.02 | 5.01 | 6.436 | |

**Table 1.** Permittivity of polar solvents

| Sample | A | B7 | B8 | B9 | B10 | B11 | B12 |
|---|---|---|---|---|---|---|---|
| peak wavelength (nm) | 517.6 | 521 | 519 | 518.8 | 518.4 | 518.6 | 517.2 |

**Table 2.**The peak wavelength of sample A and sample B7-B12.

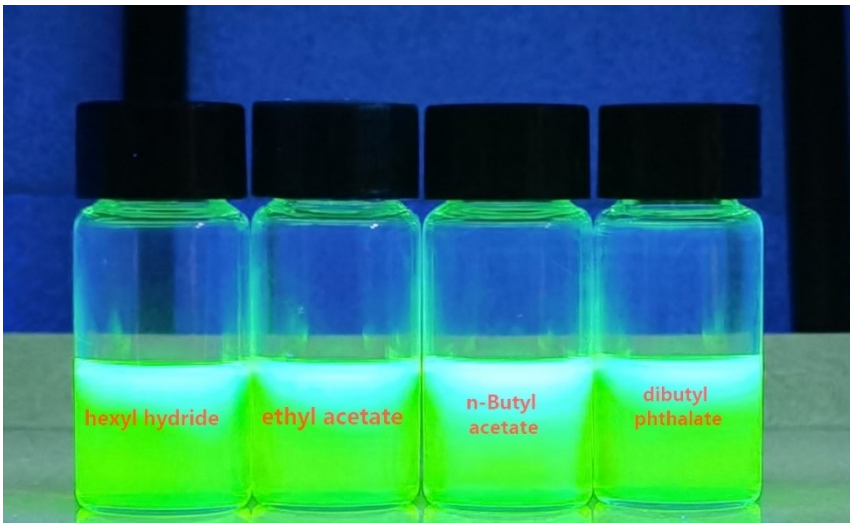


**Figure S1.** Photograph of the sample A and sample B2-B4 under UV light.

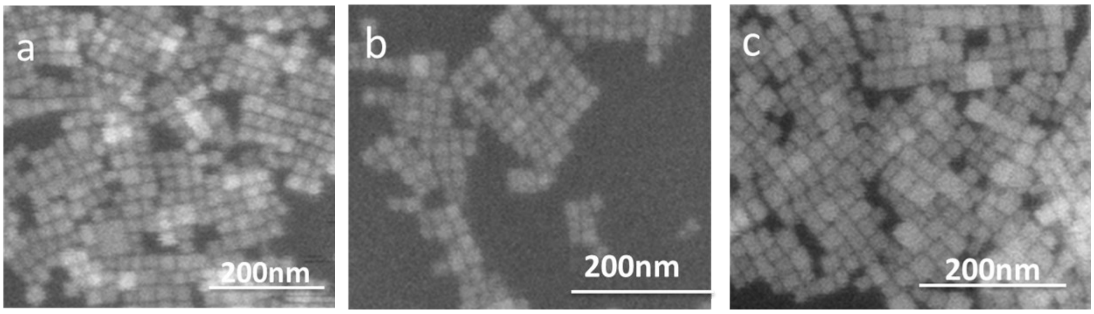


**Figure S2.** The FE-SEM images of PQDs after reacting with B2-B4.

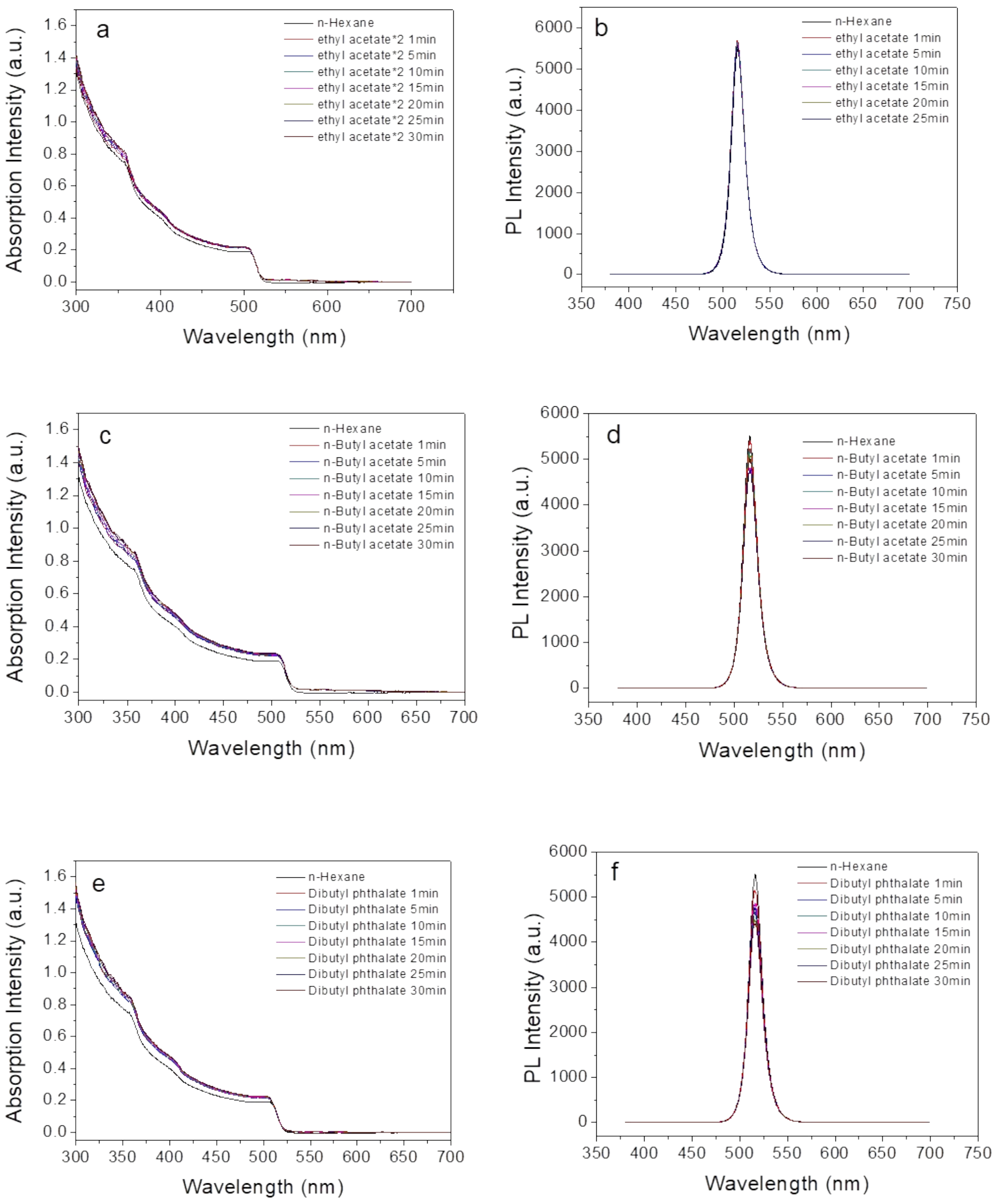


**Figure S3.** The absorption and PL spectras of sample B2-B4.

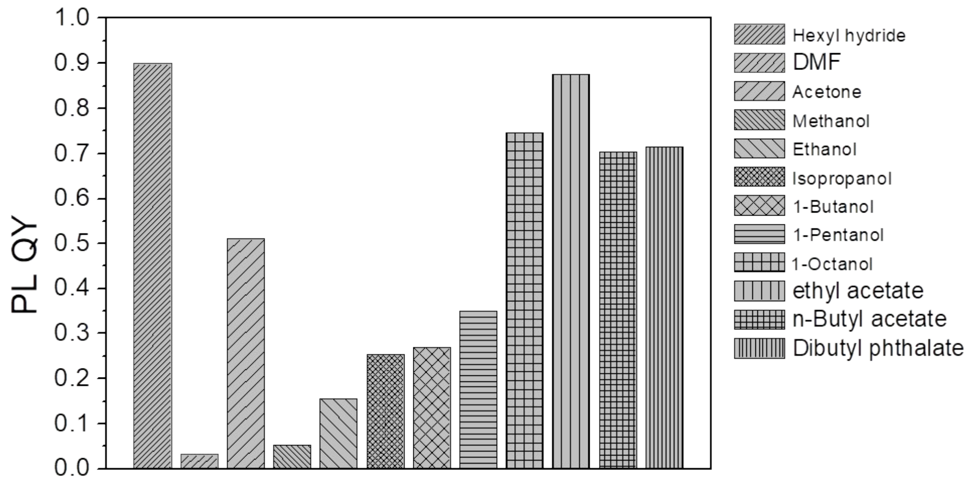


**Figure S4.** The PLQY of PQDs after adding hexyl hydride, DMF, acetone, methanol, ethanol, isopropanol, 1-butanol, 1-pentanol, octanol, ethyl acetate, n-Butyl acetate, dibutyl phthalate.

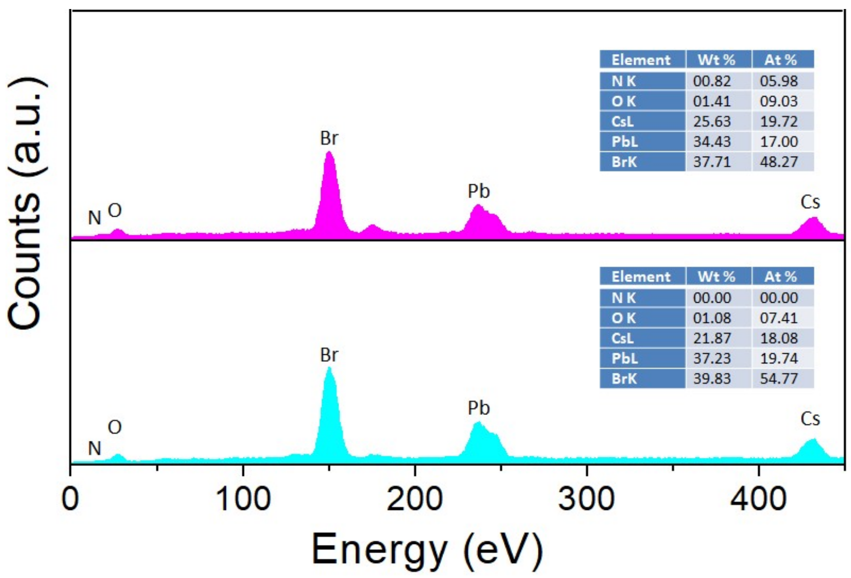


**Figure S5.** The EDS diagrams of hexyl hydride (blue) and TMEDA (pink).

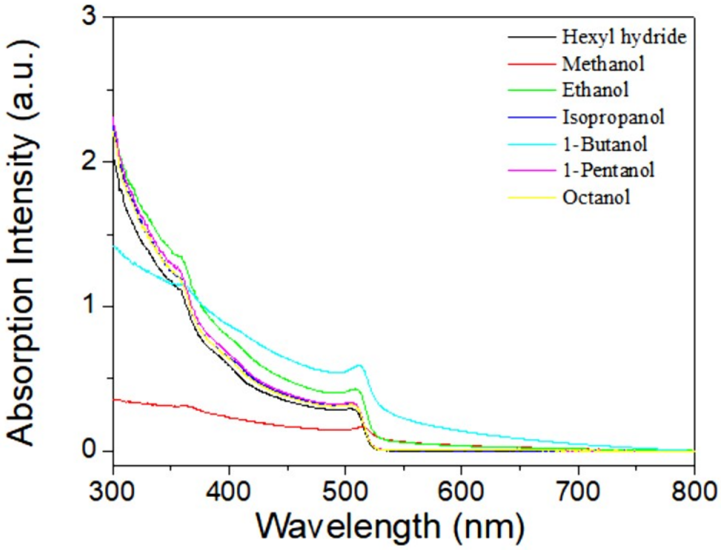


**Figure S6.** The absorption spectra of PQDs after reacting with alcohols.

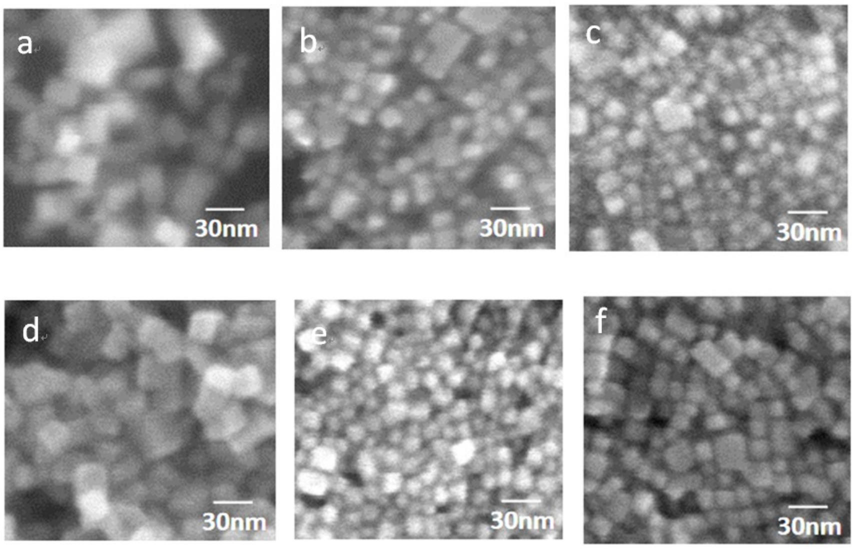


**Figure S7.** The FE-SEM images of PQDs after reacting with methanol, ethanol, isopropanol, 1-butanol, 1-pentanol, octanol.

| Polor solvent | Structure | PLQY |
|---|---|---|
| Acetone | Shedding part of the ligand molecules | 51% |
| Ethyl acetate | Shedding part of the ligand molecules | 87.58% |
| n-Butyl acetate | Shedding part of the ligand molecules | 70.34% |
| Dibutyl phthalate | Shedding part of the ligand molecules | 71.48% |
| TMEDA | Partial replacement the ligand molecules | keep a strong fluorescence reaction after standing for one year. |
| DMF | Large amounts of ligand molecules fall off and aggregate | 3.2% |
| Methanol | Large amounts of ligand molecules fall off and aggregate | 5.32% |
| Ethanol | Shedding part of the ligand molecules | 15.57% |
| Isopropanol | Shedding part of the ligand molecules | 25.3% |
| 1-Butanol | Shedding part of the ligand molecules | 26.97% |
| 1-Pentanol | Shedding part of the ligand molecules | 34.96% |
| 1-Octanol | Shedding part of the ligand molecules | 74.66% |

**Table 3.** After added different polar solvents, the changes of structural and optical properties of the PQDs.
